# Correlations Between Facial Expressivity and Apathy in Elderly People With Neurocognitive Disorders: Exploratory Study

**DOI:** 10.2196/24727

**Published:** 2021-03-31

**Authors:** Radia Zeghari, Alexandra König, Rachid Guerchouche, Garima Sharma, Jyoti Joshi, Roxane Fabre, Philippe Robert, Valeria Manera

**Affiliations:** 1 Cognition Behaviour Technology Research Unit Memory Center Université Côte d’Azur Nice France; 2 Association Innovation Alzheimer Nice France; 3 French Institute for Research in Computer Science and Automation Valbonne France; 4 Monash University Monash Australia; 5 Centre Hospitalier Universitaire de Nice Département de Santé Publique Nice France

**Keywords:** apathy, action units, assessment, ICT, facial video analysis, neurocognitive disorders, neurocognitive, facial analysis

## Abstract

**Background:**

Neurocognitive disorders are often accompanied by behavioral symptoms such as anxiety, depression, and/or apathy. These symptoms can occur very early in the disease progression and are often difficult to detect and quantify in nonspecialized clinical settings.

**Objective:**

We focus in this study on apathy, one of the most common and debilitating neuropsychiatric symptoms in neurocognitive disorders. Specifically, we investigated whether facial expressivity extracted through computer vision software correlates with the severity of apathy symptoms in elderly subjects with neurocognitive disorders.

**Methods:**

A total of 63 subjects (38 females and 25 males) with neurocognitive disorder participated in the study. Apathy was assessed using the Apathy Inventory (AI), a scale comprising 3 domains of apathy: loss of interest, loss of initiation, and emotional blunting. The higher the scale score, the more severe the apathy symptoms. Participants were asked to recall a positive and a negative event of their life, while their voice and face were recorded using a tablet device. Action units (AUs), which are basic facial movements, were extracted using OpenFace 2.0. A total of 17 AUs (intensity and presence) for each frame of the video were extracted in both positive and negative storytelling. Average intensity and frequency of AU activation were calculated for each participant in each video. Partial correlations (controlling for the level of depression and cognitive impairment) were performed between these indexes and AI subscales.

**Results:**

Results showed that AU intensity and frequency were negatively correlated with apathy scale scores, in particular with the emotional blunting component. The more severe the apathy symptoms, the less expressivity in specific emotional and nonemotional AUs was displayed from participants while recalling an emotional event. Different AUs showed significant correlations depending on the sex of the participant and the task’s valence (positive vs negative story), suggesting the importance of assessing male and female participants independently.

**Conclusions:**

Our study suggests the interest of employing computer vision-based facial analysis to quantify facial expressivity and assess the severity of apathy symptoms in subjects with neurocognitive disorders. This may represent a useful tool for a preliminary apathy assessment in nonspecialized settings and could be used to complement classical clinical scales. Future studies including larger samples should confirm the clinical relevance of this kind of instrument.

## Introduction

Apathy is one of the most common neuropsychiatric symptoms in neurocognitive disorders (NCDs) such as Alzheimer disease, Parkinson disease, Huntington disease, and vascular dementia and is prevalent in several psychiatric pathologies such as schizophrenia and major depression [1]. It can be defined as a reduction in goal-directed behavior that persists over time causing impairment in global functioning. Three dimensions of apathy have been identified, including loss/reduction in goal-directed behavior and goal-directed cognitive activity (eg, reduced interests and reduced indoor and outdoor activities), emotions (eg, emotional blunting), and social interactions (eg, reduced interactions with family members and friends) [2]. Apathy is a debilitating symptom: it significantly decreases the quality of life of patients with NCD and their caregivers and increases the risk for institutionalization in outpatients [3] and mortality for nursing home residents (even after controlling for depression) [4]. Apathy has also been associated with faster cognitive and functional decline [5,6]. A recent study suggested that apathy significantly increases the risk of developing dementia, resulting in a loss of autonomy in activities of daily living [7]. As apathetic persons interact less with their surroundings, show less interests in their family and in leisure activities, they are significantly less stimulated, which may increase the progression of NCD. Critically, preliminary evidence suggests that interventions targeting apathy in people with mild cognitive impairment (through repetitive transcranial magnetic stimulation) may be effective in improving global cognitive functioning [8], thus suggesting that identifying apathy early in disease progression and putting in place early treatment options could offer new opportunities for dementia prevention [9].

Currently, apathy is assessed by using various clinical scales or questionnaires (including self-reports and scales completed by caregivers and/or the clinician; see Radakovic et al [10] for a review), which allow for quantification of apathy symptoms over continuous scales. Furthermore, apathy can be assessed using the Apathy Diagnostic Criteria (ADC), which allow for classification of patients as apathetic versus nonapathetic based on the observed symptomatology [2]. All these instruments suffer from the risk of bias resulting from the assessor’s subjectivity [11]. For instance, self-report clinical scales rely on the patient’s ability to recall a change in their activities, interests, or emotional reactivity, which might be difficult considering that this requires preserved memory and insight to some extent. Similarly, the clinician may have access only to limited information concerning the patient’s changes in everyday activities, thus resulting in underestimation of apathy [11].

Another obstacle that can emerge is that apathy can often be misdiagnosed as depression because of frequent comorbidities such as fatigue and anhedonia and a considerable overlap in key symptoms such as diminished interest, psychomotor retardation, or social withdrawal [12-14]. However, apathy and depression can be dissociated based on emotional deficits. Indeed, the emotion dimension in apathy can be described as a blunted affect whereas depression is characterized by the presence of negative emotions and sadness. Early differential diagnosis is of great importance since treating apathy with antidepressants can lead to aggravation of symptoms [2,11]. Therefore, there is an urgent need to detect apathy more objectively at early stages using objective, noninvasive methods to provide timely treatment and prevent it from aggravating cognitive symptoms. There is today a growing interest in finding new sensitive measures to detect behavioral aspects of dementia as they may represent a heavier burden for caregivers than the cognitive dysfunctions themselves [15,16].

New information and communication technologies can provide objective and more sensitive measures of human behaviors and have been recommended for neurocognitive disorders and apathy [17,18]. The behavioral correlates of apathy have been investigated through oculomotor movement using eye tracking [19], global activity or sleep disturbances using accelerometers [20,21], voice features [22,23], and facial expressivity [24-26]. The additional value of combining multimodal measures has been demonstrated before in depression by merging audio features with facial activity [27-29]. Associating these new markers with other recent methods such as ecological momentary assessment [30,31], which allows patients to report on symptoms remotely, could represent the future of more naturalistic psychiatric evaluations. These techniques allow the tracking of changes in mood and cognition continuously leading to timely prevention of further decline.

Today, facial expressivity, which may be altered in apathetic subjects, can be measured automatically by means of computer vision-based facial analysis methods. The Facial Action Coding System allows detection of facial behavior by identifying specific facial muscle movements called action units (AUs) [32]. Some AUs are specifically linked to emotions such as AU 4 (cheek raiser) and 12 (lips puller, smile) being linked to joy. Some AUs are mainly associated with positive emotions, while others are more associated with negative emotions (see Table 1 for a description of AUs). Girard and colleagues [33] found that automatic facial expression analysis was consistent with manual coding, validating its use in clinical research. In their study, they analyzed AUs one by one to extract a pattern linked to depression. Seidl and colleagues [26] studied the association of cognitive decline and facial expressivity. The results showed apathy moderated the effect of cognitive decline on facial expression in Alzheimer disease and was significantly correlated to decreased general and specific facial expressivity.

**Table 1:** Action unit descriptions [32] and emotion valence [34].

AU^a^ number	FACS^b^ name	Emotion valence related
1	Inner brow raiser	–^c^
2	Outer brow raiser	+^d^
4	Brow lowerer	–
5	Upper lid raiser	–
6	Cheek raiser	+
7	Lid tightener	+/–^e^
9	Nose wrinkler	–
10	Upper lip raiser	–
11	Nasolabial deepener	=^f^
12	Lip corner puller	+
14	Dimpler	+
15	Lip corner depressor	–
17	Chin raiser	=
20	Lip stretcher	–
23	Lip tightener	+/–
25	Lips part	+
26	Jaw drop	+
45	Blink	=

^a^AU: action unit.

^b^FACS: Facial Action Coding System.

^c^Negative valence.

^d^Positive valence.

^e^Either positive or negative valence.

^f^Neutral.

In a previous pilot study, we aimed to identify facial behavior associated with apathy through automated video analysis and investigate how facial variations (expression and movement) could help characterize this symptom [24]. Our algorithm was able to classify subjects as apathetic versus nonapathetic with 84% accuracy. To validate the deep learning algorithm, we relied on the leave-one-out cross-validation technique, which consists of training a model using n–1 available subjects and validate using the remaining subject. In another research work, we employed a different approach with the same dataset [25]. Here again we used deep learning techniques to build models but added audio features to the video ones. The final model was 76% accurate in discriminating apathetic versus nonapathetic subjects.

Despite these promising findings, the problem of using deep learning techniques for such studies is that the generally low number of included subjects makes it difficult to allow for a generalization of the built models. In addition, while correct classification of apathetic versus nonapathetic subjects is an important challenge, from a clinical point of view it is crucial to precisely understand which features are more sensitive for apathy assessment and investigate links between these features and the degree of apathy severity as well as its subdomains, as measured by continuous scales.

Therefore, we aim with this exploratory study to better understand the quantitative relationship between facial expressivity and apathy and its subdomains (emotional blunting, loss of initiation, loss of interest) independently of depression and level of cognitive decline, which have been previously shown to affect emotional expressivity. For this, we included a larger sample of participants from which we correlated basic facial AU activation and intensity with apathy scale scores.

We hypothesize that the higher the apathy score of a participant, the lower and less intense would be his facial expressivity especially for AUs involved in positive or negative emotions [34].

## Methods

### Participants

A total of 63 subjects were included in the study; 7 participants had subjective memory complaints and the rest were diagnosed with NCDs [35] (39 mild and 17 major), including 11 subjects with Alzheimer disease, 22 subjects with vascular dementia, and 11 subjects with affective disorders. Participants were recruited from the Memory Center of Nice University Hospitals, France, and from the Cognition Behavioral Technology research lab of the Université Cote d’Azur in the context of motivation activation research protocol. Participants with mild NCD were previously followed at the Memory Center. Participants were not included if they had sensory or motor impairments interfering with the protocol completion. For each participant, clinicians assessed the global level of cognitive impairment using the Mini-Mental State Examination (MMSE) [36], the presence of depression using the Neuropsychiatric Inventory (NPI) [37], the presence of apathy using the ADC [2], and the severity of apathy symptoms using the Apathy Inventory (AI) [38]. The AI is divided into three subscales: loss of initiation (AI-initiation), loss of interest (AI-interest), and emotional blunting (AI-affect). The NPI depression score is calculated by reporting the intensity (1=mild, 2=moderate, 3=severe) and frequency (1=rarely to 4=very often). The demographic and clinical profiles of participants are presented in Table 2. Additional demographic features (including comparisons between apathetic versus nonapathetic subjects based on the ADC) are reported in Multimedia Appendix 1.

**Table 2:** Sociodemographic, cognitive, and behavioral variables of study participants.

Characteristic	Total (n=63), mean (SD)	Females (n=38), mean (SD)	Males (n=25), mean (SD)	Cohen *d*
Age in years	72.95 (8.40)	72.66 (8.89)	73.40 (7.76)	0.09
MMSE^a^	24.06 (3.84)	23.68 (4.09)	24.64 (3.43)	0.25
NPI^b^ depression	1.17 (1.95)	1.13 (2.03)	1.24 (1.85)	0.06
AI^c^ affect	0.33 (0.74)	0.24 (0.68)	0.48 (0.82)	0.33
AI initiation	1.32 (1.47)	1.00 (1.38)	1.80 (1.50)	0.56
AI interest	1.17 (1.33)	0.92 (1.30)	1.56 (1.29)	0.49
AI total	2.81 (3.03)	2.11 (2.83)	3.88 (3.07)	0.61

^a^MMSE: Mini-Mental State Examination.

^b^NPI: Neuropsychiatric Inventory.

^c^AI: Apathy Inventory.

### Ethics

The study was performed as defined in the Declaration of Helsinki. The protocol was approved by the ethics committee (Comité de Protection de Personnes—CPP Est III, France; MoTap: RCB ID No. 2017-A01366-4). Informed written consent was obtained from all participants before the study.

### Free Emotional Speech Task

Participants were asked to recall a positive and negative event of their life in maximum 1 minute. This free speech task requires only a low cognitive load and is supposed to trigger an emotional response. Participants were audio and videorecorded using a tablet device in a quiet room of the Resources and Research Memory Center at the Claude Pompidou Institut in Nice. The tablet was facing the subjects but not hiding the investigator, allowing a more natural interaction. In a previous study, we have analyzed the simple audio files and found that certain voice features were associated with apathy presence [23].

### Video Features Extraction

For the features extraction we used OpenFace 2.0 software, which is an open-source facial behavior analysis toolkit [39]. OpenFace is an implementation of face recognition with deep neural networks. OpenFace allows detection of a single or multiple faces in an image using pretrained models. Many basic features can be extracted such as facial landmarks, eyes, and head positions. Higher level features are also provided by OpenFace in a video: head direction and movements, eye gaze estimations, and expressed emotions. Emotions are mainly computed by combining several AUs. Since the accuracy of detected emotions is highly related to the datasets used for training the neural networks and since we are working with a small dataset of elderly people that doesn’t allow us to retrain the model, the obtained accuracy on detected emotions is quite unsatisfying. We then investigated the use of AUs as middle-level features to detect apathy. We use here the 17 main computed AUs by OpenFace: 1, 2, 4, 5, 6, 7, 9, 10, 12, 14, 15, 17, 20, 23, 25, 26, and 45 (see Table 1). For each frame of the recorded videos, OpenFace provides AU intensity and presence. We then calculated 2 measures for each AU in both videos: the average intensity and activation of each AU in each video. Intensity ranged from 0 (absent) to 1 (present at minimum intensity) to 5 (present at maximum intensity), with continuous values in between. The mean intensity for each AU was calculated as the average score across all the video frames. To obtain a more general measure of AU activation, we also computed for each subject the mean activation and intensity across all AUs.

### Statistical Analysis

Statistical analyses were performed using SPSS Statistics version 23.0.0 for Mac software (IBM Corporation) and R version 3.6.3 (R Foundation for Statistical Computing). To investigate the linear relationships between apathy and facial expressivity, we performed partial linear correlations between AU activation and intensity and the AI subscales using depression (NPI depression) and level of cognitive impairment (MMSE score) as covariates for both the positive and negative story. As most of the clinical scales were not normally distributed (as indexed by Shapiro-Wilks tests), Spearman rho correlations were employed. We wanted to control for the effect of cognitive impairment as it might have an impact on the task itself to recall an emotional event. Similarly, we wanted to control for the effects of depressive symptoms. In the analyses run on each specific AU, we separated males and females as studies have found that women and men can express their emotions differently [40-42].

## Results

### Descriptive Analysis

A total of 63 subjects (38 females and 25 males) were included in the study. The demographic and clinical profiles of participants are presented in Table 2. Sociodemographic features of apathetic and nonapathetic subjects and correlations among the different clinical scales are reported in Multimedia Appendix 1.

The average activation frequency and intensity for each AU are reported in Multimedia Appendix 1. In the positive story, the average global intensity of all AUs was 0.52 (SD 0.15) and the average global frequency was 0.29 (SD 0.07). In the negative story, the average global intensity was 0.51 (SD 0.28) and global frequency 0.28 (SD 0.08). Average presence and intensity for each AU for males and females are presented in Multimedia Appendix 1. In the negative story, AU 7 was the most intense facial expression in the whole sample (mean 1.60 [SD 0.84] on a 0 to 5 scale) followed by AU 10 (mean 1.02 [SD 0.47]) and AU 4 (mean 0.45 [SD 0.57]). AU 5 was the most frequently seen in the video (mean 0.68 [SD 0.30]) followed by AU 4 (mean 0.37 [SD 0.34) and AU 7 (mean 0.37 [SD 0.31]). In the positive story, AU 7 was also the most intense (mean 1.68 [SD 0.86]) followed by AU 10 (mean 1.1 [SD 0.50]) and AU 6 (mean 0.85 [SD 0.53]). AU 5 was the most frequently seen (mean 0.63 [SD 0.33]) followed by AU 7 (mean 0.40 [SD 0.32]) and AU 23 (mean 0.39 [SD 0.29]).

### AU Correlations to Scales

#### Global Scales

A general analysis considering the global activation and intensity across AUs revealed a small but significant negative partial correlation between global AU intensity to AI-affect score in the negative story (r_s(61)_=–0.26, *P*=.04), suggesting that more apathetic participants showed lower AU intensity in the negative story. No other significant linear correlation was found between the apathy scales and global activation.

#### Single AUs

Spearman partial correlations between each AU and apathy scale score controlled by the NPI depression and MMSE scores and divided by sex are presented in Table 3. Only significant correlations are reported. All correlations are presented in Multimedia Appendix 1.

For females, we found significant negative partial correlations between AUs and apathy, predominantly with the AI-affect subscale score, in both negative and positive stories (all of them of medium effect size, rho ranging from –0.33 to –0.47). All correlations were negative. The more severe the AI-affect score, the less frequent or less intense the AU. Specifically, significant correlations were found for AU 1, 2, 9, 10, 12, 25, and 45. In the positive story, the mean activations of AU 10 and 25 were correlated to AI-affect score. In the negative story, the mean activations of AU 1, 2, 9, 10 12, and 45 were correlated to AI-affect scores. The mean activations of AU 10 and 12 were correlated to AI-total score, and the mean activation of AU 10 was correlated to AI-initiation score.

For males, significant correlations were found for AU 1, 12, 14, 15, 17, 20, and 45. All of these correlations were negative, with effect sizes ranging from medium to large (rho ranging from –0.42 to –0.63). In the positive story, the mean intensity of AU 1 was correlated to AI-initiation score, 12 to AI-interest score, 14 to AI-total score, and 17 was correlated to AI-affect score. The mean activation of AU 14 was correlated to AI-initiation, AI-interest, and AI-total scores. The mean activation of AU 45 was correlated to AI-interest and AI-total scores. In the negative story, the mean intensity of AU 1 was correlated to AI-initiation and AI-total scores. The mean intensities of AU 15, 17, and 20 were correlated to AI-affect score. The mean activation of AU 28 was correlated to AI-initiation and NPI-apathy score.

**Table 3:** Spearman rho correlations between action units and apathy scales for males and females.

Characteristics, AU^a^				AI^b^-affect	AI-initiation	AI-interest	AI-total
**Female**							
	**Negative story**						
		**Mean activation**					
			Inner brow raiser	–0.340	—^c^	—	—
			Outer brow raiser	–0.422	—	—	—
			Nose wrinkler	–0.382	—	—	—
			Upper lip raiser	–0.350	–0.426	—	–0.337
			Lip corner puller		—	—	–0.350
			Blink	–0.349	—	—	—
		**Mean intensity**					
			Inner brow raiser	–0.361	—	—	—
			Nose wrinkler	–0.470	—	—	—
	**Positive story**						
		**Mean activation**					
			Upper lip raiser	–0.461	—	—	—
			Lips part	–0.333	—	—	—
**Male**							
	**Negative story**						
		**Mean activation**					
			Lip stretcher	–0.474	—	—	—
		**Mean intensity**					
			Inner brow raiser	—	–0.432	—	–0.426
			Lip corner depressor	–0.628	—	—	—
			Chin raiser	–0.510	—	—	—
			Lip stretcher	–0.472	—	—	—
	**Positive story**						
		**Mean activation**					
			Dimpler	—	–0.501	–0.625	–0.503
			Blink	—	—	–0.428	–0.466
		**Mean intensity**					
			Inner brow raiser	—	–0.472	—	—
			Lip corner puller	—	—	–0.418	—
			Dimpler	—	—	—	–0.416
			Chin raiser	–0.437	—	—	—

^a^AU: action unit.

^b^AI: Apathy Inventory.

^c^Not applicable.

## Discussion

### Principal Findings

In this exploratory study we aimed to verify whether facial expressivity, assessed using automatic video analysis, correlates with apathy severity in elderly subjects with NCDs. Specifically, we aimed to explore which particular AUs, intensity and activation frequency, are associated with apathy and its different subdomains in male and female subjects, employing 2 different emotion-related tasks (telling a positive and a negative story).

To assess apathy, we used the AI scale (clinician version) and its subdomains reduced affect, loss of interest, and loss of initiation. We extracted AUs from videorecordings of participants while they performed an emotional task consisting of recalling a positive and a negative event from their life. Studies have found that women and men can express their emotions differently [40-42]. Thus, we separated male and female participants for the analyses. We hypothesized that the AUs implicated in positive and negative emotions (joy, sadness, disgust, anger, surprise) would correlate with apathy levels. Specifically, the more important the apathy symptomatology, the lower the emotional activation/intensity. We classified AUs with emotional valence (positive, negative, neutral) according to Haines et al [34].

Partial correlations (controlling for depressive symptoms and level of cognitive impairment) showed that, overall, apathetic participants showed lower average AU intensity in the negative story. This result suggests that more apathetic subjects had less intense facial expressivity compared with less apathetic subjects, at least while telling a negative event. At the level of single AU analysis, several AUs were significantly less expressed with more pronounced apathy symptoms in the upper region (inner and outer brow raiser) and lower region (nose and lip movement) of the face. Moreover, the relevant AUs were different depending on sex and the task’s emotional valence.

For instance, for men, AU 12 (smile) and 14 (dimpler), both considered positive AUs, were significantly less expressed the more important the behavioral and cognitive dimensions of apathy in the positive story. AU 15 (lip corner depressor) and 20 (lip stretcher), both considered negative AUs, correlated significantly with affect dimension in the negative story. AU 1 (inner brow raiser), involved in sadness, anger and fear, was less expressed in both stories the more severe the initiation symptoms. For women, in the positive story, AU 1 (inner brow raiser) intensity and 4 (brow lowerer) frequency (both involved in sadness) were significantly correlated to AI-affect in the negative story. Thus, some of the expected facial expressions are significantly less expressed in more apathetic subjects. For men, this was observed in both positive and negative storytelling. For women, this was mainly observed in the negative story. For women, AU 25 (lip part), involved in talking, was significantly less frequent in the positive story. This is in line with the symptomatology of apathetic subjects being less talkative [23]. Different AUs were correlated to apathy scales depending on the task’s emotional valence. In the negative story, mainly negative valence AUs were correlated to apathy symptoms for men. Both positive and negative AUs showed significant correlations for women. In the positive story, positive AUs (12 and 14) and neutral AUs (17 and 45) were significantly less expressed for men. For women, only two AUs (10 and 25) showed significant correlations, one of negative and one of positive valence. Our findings suggest that the more severe the apathy symptoms, the less expressed are the AUs expected for the task’s valence.

### Correlations With the Affect Apathy Dimension

For both men and women in the negative story, the majority of AUs were associated with the affect subdomain. AI-affect (blunted affect) is mainly assessed with facial emotional responses in clinical interviews, which could explain this finding. Depending on the patient’s emotional responsiveness in the interaction, the clinician will infer the presence of a potential emotional blunting. This is one of the main biases in the evaluation of apathy due to a lack of objectivity (clinician can miss cues) and context (patients are in a particular environment that does not translate the way they experience emotions in their daily lives). Similar studies found that flattened affect in schizophrenic or depressed patients is reflected in decreased spontaneous facial expression compared with control groups [43]. Furthermore, they found that depressed patients showed even less facial expressivity compared with schizophrenic subjects.

Interestingly, blunted affect was the subdomain of apathy most linked to lack of facial expressivity for women. This could be explained by the hypothesis that women tend to express their emotions more [40,41,44]. Studies have shown that emotional expressivity and emotional memory retrieval are different depending on sex [45,46]. Men experience emotions more strongly whereas women tend to express them more. Another explanation would be that women are expected to express more happiness and sadness, and a lack of facial expressivity due to apathy would be identified more quickly than for men who are less expected to display these emotions [47]. Future studies should investigate the link between subdomains of apathy and sex. We found that for women, AU 9 (nose wrinkler, involved in disgust) intensity was significantly correlated with AI-affect in the negative story. In one study, the authors predicted the sex of a person simply from their emotional expressions of happiness and disgust but not from expressions like sadness and surprise [41]. This AU is expected to be more pronounced and intense for women but seems significantly less expressed in people with apathy.

### Correlations With the Loss of Interest and Lack of Initiation Dimensions

Only a few AUs were linked to loss of interest and lack of initiation. For women, only AU 10 was linked to lack of initiation, and none to loss of interest. However, for men, in the positive story, all the significant correlations to AUs (such as 1 and 14) were linked to either lack of initiation or loss of interest. One explanation could be that the positive condition triggered memories that might have involved activities such as gatherings with family or friends, eliciting less intense positive emotions the more severe the apathy symptoms. Emotional valence (positive, negative, and neutral) and arousal of an event will have an impact on the way emotions are stored and retrieved in autobiographical memory [48]. Positive memories are retrieved with richer details supposedly due to a bonding social role, while negative memories would have more of a survival role, so the events are not repeated. In the positive story, women often recalled the birth of a child or a wedding day while men recalled more diverse events.

Our results, despite being preliminary, are in line with previous research showing that besides cognitive deficits, apathy was significantly correlated with decreased overall and specific facial expression [26]. In depression, similar results have been found [33]. Girard et al [33] compared specific AUs to depression severity and found that depressed people would smile less and express the emotion of contempt more. In apathy, the AUs involved in these emotions were all decreased for the male sample. However, different AUs were involved for the female sample, which underlines how important it is to consider sex differences in the study of emotional expressivity. Overall, apathy seems to have an impact on global facial expressivity and thus, can be detected by automated video analysis. Nevertheless, at this stage it remains a rather sensitive but not specific tool for detecting variations in emotional facial expression (which in turn can be an indicator for apathy or depression or negative symptoms, etc). Peham et al [49] studied facial emotional behavior of female subjects suffering from mental disorders (personality disorder, depression, anxiety, eating disorder, etc) during a clinical interview and found no distinctive patterns that were disease specific.

In depression detection, increasingly research efforts have been placed on merging audio and video features with encouraging results, demonstrating that when both modalities are combined more precise assessments can be obtained [27]. We previously demonstrated that, simply by extracting and analyzing voice features from the free emotional tasks, apathy prediction can be quite accurate [22,23]. Therefore, it can be assumed that in a next step, the fusion of audio and video features will improve on our current results, supporting the long-term goal of validating such technology for use in daily clinical practice.

### Limitations

The main study limitations are the relatively small sample size, coupled with the rather high number of statistical tests performed. As this study was exploratory, we did not employ statistical corrections for multiple comparisons, which could increase the probability of making type I errors. This is one of the first studies that analyzes correlations between facial expressivity and apathy, and these results could be used to formulate more precise hypotheses on specific AU involvement, to be tested with more robust statistical methods in future studies. Another issue is that this study relied on clinicians to rate levels of apathy and other neuropsychiatric scales. Future research should employ multiple types of scales such as self-administered scales in order to understand if there is a gap between how the patient feels in his daily life and what the clinician is observing. Combining ecological momentary assessment with these novel measures (facial behavior, voice parameters, activity monitoring, etc) and biomarkers (magnetic resonance imaging) should also be included to ensure covering all types of assessments available since there are no existing gold standard for apathy scales [11,50]. More variables should be considered such as fatigue and anhedonia, as they both are related to apathy [14,51].

### Conclusion

Overall, it can be concluded that computer vision-based facial analysis showed promising results in detecting blunted affect and global apathy in neurocognitive disorders. These preliminary results should be corroborated by further studies including a larger sample size, allowing researchers to test a reduced number of relevant hypotheses and apply corrections for multiple comparisons. As the effect size found in our study was medium to large, reduced facial expressivity may represent a promising proxy for emotional blunting. Specifically, as hypothesized, our results suggested that the presence of AUs relating to positive emotions is particularly relevant to assess apathy during the positive storytelling, while the presence of AUs relating to negative emotions may be relevant during negative storytelling, especially for men. Women may show a wider range of emotions in both positive and negative storytelling, thus suggesting the interest of assessing AUs related to both positive and negative emotions in both stories.

More research is needed to identify specific facial expressions associated with apathy and combine this method with other technologies such as automatic speech analysis and eye tracking to provide additional information for differential diagnosis. Identifying multimodal digital biomarkers (eg, voice features, activity patterns, eye paths, facial expression) and combining them with ecological momentary assessment could be the future of neuropsychiatric and cognitive assessment, allowing early detection of changes and therefore better adapted treatment [30]. These technologies could facilitate continuous monitoring to prevent relapses in depression, psychotic crisis in schizophrenia, or the detection of early signs of cognitive deficits and behavioral changes in neurocognitive disorders.

## Appendix

Multimedia Appendix 1 Auxiliary tables.

## Abbreviations

ADC: Apathy Diagnostic Criteria
AI: Apathy Inventory
AU: action unit
MMSE: Mini-Mental State Examination
NCD: neurocognitive disorder
NPI: Neuropsychiatric Inventory
UCA-JEDI: Cote d’Azur University: Joint, Excellent, and Dynamic Initiative
