# Supplementary material for: Correlations Between Facial Expressivity and Apathy in Elderly People With Neurocognitive Disorders: Exploratory Study
Source: JMIR Form Res. 2021 Mar 31;5(3):e24727. doi: 10.2196/24727 (PMC8047819; doi:10.2196/24727)
Supplement: Multimedia Appendix 1 [file formative_v5i3e24727_app1.docx]

Multimedia Appendix 1

1. **Between group comparison: Variable Apathy (ADC)**
2. **Scales correlations**
3. **Summary of Means**
4. **Partial correlations: male sample**
5. **Partial correlations: female sample**
6. **ST1. Between group comparison**

|  | **Total**  **(n = 63)** | | **NA**  **(n= 38)** | | **A**  **(n = 24)** | |  | |
| --- | --- | --- | --- | --- | --- | --- | --- | --- |
|  | Mean | (SD) | Mean | (SD) | Mean | (SD) | p* | |
| *Age* | 72,95 | 8,40 | 71,62 | 8,038 | 75,13 | 8,699 | ,163 | |
| *MMSE* | 24,06 | 3,84 | 25,08 | 3,572 | 22,42 | 3,764 | ,006 | |
| *NPI anxiety* | 2,54 | 2,49 | 2,41 | 2,572 | 2,75 | 2,382 | ,411 | |
| *NPI depression* | 1,17 | 1,95 | ,67 | 1,402 | 2,00 | 2,414 | ,011 | |
| *NPI Apathy* | 2,57 | 3,22 | ,49 | ,970 | 5,96 | 2,662 | ,000 | |
| *NPI total* | 9,10 | 6,87 | 5,67 | 4,257 | 14,67 | 6,716 | ,000 | |
| *AI Affect* | 0,33 | 0,74 | ,08 | ,270 | ,75 | 1,032 | ,001 | |
| *AI Initiation* | 1,32 | 1,47 | ,38 | ,673 | 2,83 | 1,090 | ,000 |  |
| *AI Interest* | 1,17 | 1,33 | ,41 | ,785 | 2,42 | 1,060 | ,000 |  |
| *IA Total* | 2,81 | 3,03 | ,82 | 1,073 | 6,04 | 2,293 | ,000 |  |

Mean (standard deviation); Between group comparison: Wilcoxon-Mann- Whitney test. NA, No Apathy; A, Apathy; MMSE, Mini-Mental State Examination; AI, Apathy Inventory; AI-Interest, AI domain Interest; AI-Initiation, AI domain Initiative; AI-Affect, AI domain affective; NPI, Neuropsychiatric Inventory; NPI-Apathy, NPI domain apathy; NPI-Depression, NPI domain depression; NPI-Anxiety, NPI domain anxiety.

1. **ST2. Scales correlations. Rho spearman**.

|  | age | MMSE | NPI anxiété | NPI dépression | NPI apathie | NPI total | AI affect | AI initiation | AI  interest | AI total |
| --- | --- | --- | --- | --- | --- | --- | --- | --- | --- | --- |
| *age* | 1,000 |  |  |  |  |  |  |  |  |  |
| *MMSE* | -,316^*^ | 1,000 |  |  |  |  |  |  |  |  |
| *NPI anxiety* | ,149 | -,251^*^ | 1,000 |  |  |  |  |  |  |  |
| *NPI depression* | ,044 | -,216 | ,245 | 1,000 |  |  |  |  |  |  |
| *NPI apathy* | ,252^*^ | -,372^**^ | ,115 | ,358^**^ | 1,000 |  |  |  |  |  |
| *NPI total* | ,252^*^ | -,245 | ,474^**^ | ,495^**^ | ,734^**^ | 1,000 |  |  |  |  |
| *AI affect* | -,177 | ,060 | -,099 | ,148 | ,401^**^ | ,239 | 1,000 |  |  |  |
| *AI initiation* | ,216 | -,363^**^ | ,057 | ,405^**^ | ,817^**^ | ,669^**^ | ,397^**^ | 1,000 |  |  |
| *AI interest* | ,072 | -,248^*^ | ,113 | ,354^**^ | ,767^**^ | ,576^**^ | ,356^**^ | ,623^**^ |  |  |
| *AI total* | ,132 | -,313^*^ | ,098 | ,431^**^ | ,883^**^ | ,684^**^ | ,499^**^ | ,892^**^ | ,874^**^ |  |

* p < 0,05 ** p < 0,01

MMSE, Mini-Mental State Examination; AI, Apathy Inventory; AI-Interest, AI domain Interest; AI-Inititation, AI domain Initiative; AI-Affect, AI domain affective; NPI, Neuropsychiatric Inventory; NPI-Apathy, NPI domain apathy; NPI-Depression, NPI item depression; NPI-Anxiety, NPI item anxiety.

1. ST3. Mean tables

Whole sample’s table means of facial AU Mean activation and mean intensity in the negative and positive story (n=63). Mean (SD).

| Negative Story | | | | |  | Positive story | | | | |
| --- | --- | --- | --- | --- | --- | --- | --- | --- | --- | --- |
| AU | Average intensity | | Average activation | |  | AU | Average intensity | | Average activation | |
| 1 | 0,345 | (0,167) | 0,273 | (0,101) |  | 1 | 0,31 | (0,149) | 0,2816 | (0,101) |
| 2 | 0,129 | (0,056) | 0,321 | (0,122) |  | 2 | 0,14 | (0,088) | 0,3121 | (0,127) |
| 4 | 0,745 | (0,572) | 0,368 | (0,337) |  | 4 | 0,744 | (0,593) | 0,3531 | (0,341) |
| 5 | 0,075 | (0,025) | 0,68 | (0,297) |  | 5 | 0,074 | (0,028) | 0,6273 | (0,331) |
| 6 | 0,74 | (0,448) | 0,219 | (0,239) |  | 6 | 0,846 | (0,528) | 0,2722 | (0,276) |
| 7 | 1,603 | (0,843) | 0,365 | (0,312) |  | 7 | 1,683 | (0,856) | 0,4045 | (0,319) |
| 9 | 0,108 | (0,046) | 0,123 | (0,107) |  | 9 | 0,106 | (0,048) | 0,118 | (0,095) |
| 10 | 1,017 | (0,474) | 0,267 | (0,279) |  | 10 | 1,098 | (0,502) | 0,3201 | (0,296) |
| 12 | 0,354 | (0,358) | 0,153 | (0,23) |  | 12 | 0,431 | (0,385) | 0,1734 | (0,223) |
| 14 | 0,597 | (0,46) | 0,293 | (0,304) |  | 14 | 0,617 | (0,436) | 0,3008 | (0,298) |
| 15 | 0,312 | (0,155) | 0,283 | (0,145) |  | 15 | 0,295 | (0,158) | 0,2841 | (0,125) |
| 17 | 0,696 | (0,216) | 0,249 | (0,098) |  | 17 | 0,629 | (0,207) | 0,2661 | (0,1) |
| 20 | 0,199 | (0,091) | 0,251 | (0,121) |  | 20 | 0,193 | (0,085) | 0,2367 | (0,113) |
| 23 | 0,226 | (0,102) | 0,365 | (0,282) |  | 23 | 0,214 | (0,109) | 0,386 | (0,287) |
| 25 | 0,583 | (0,198) | 0,294 | (0,071) |  | 25 | 0,608 | (0,207) | 0,295 | (0,085) |
| 26 | 0,64 | (0,259) | 0,234 | (0,089) |  | 26 | 0,635 | (0,217) | 0,2332 | (0,09) |
| 45 | 0,321 | (0,118) | 0,292 | (0,095) |  | 45 | 0,301 | (0,109) | 0,295 | (0,095) |
| total | 0,511 | (0,133) | 0,282 | (0,078) |  | total | 0,525 | (0,146) | 0,2884 | (0,076) |

Female sample table means of facial AU mean activation and mean intensity in the negative and positive story (n=37). Mean (SD).

| Negative Story | | | | |  | Positive story | | | | |
| --- | --- | --- | --- | --- | --- | --- | --- | --- | --- | --- |
| AU | Average intensity | | Average activation | |  | AU | Average intensity | | Average activation | |
| 1 | 0,377 | (0,162) | 0,29 | (0,101) |  | 1 | 0,324 | (0,146) | 0,28 | (0,108) |
| 2 | 0,141 | (0,061) | 0,332 | (0,133) |  | 2 | 0,149 | (0,098) | 0,311 | (0,145) |
| 4 | 0,812 | (0,581) | 0,395 | (0,339) |  | 4 | 0,774 | (0,604) | 0,377 | (0,353) |
| 5 | 0,074 | (0,023) | 0,645 | (0,271) |  | 5 | 0,072 | (0,022) | 0,573 | (0,338) |
| 6 | 0,752 | (0,458) | 0,238 | (0,248) |  | 6 | 0,874 | (0,575) | 0,309 | (0,301) |
| 7 | 1,541 | (0,834) | 0,43 | (0,316) |  | 7 | 1,617 | (0,896) | 0,473 | (0,329) |
| 9 | 0,108 | (0,043) | 0,115 | (0,093) |  | 9 | 0,106 | (0,051) | 0,118 | (0,096) |
| 10 | 0,985 | (0,499) | 0,253 | (0,275) |  | 10 | 1,091 | (0,529) | 0,316 | (0,269) |
| 12 | 0,401 | (0,374) | 0,166 | (0,232) |  | 12 | 0,51 | (0,408) | 0,212 | (0,257) |
| 14 | 0,61 | (0,505) | 0,295 | (0,333) |  | 14 | 0,636 | (0,468) | 0,312 | (0,323) |
| 15 | 0,288 | (0,141) | 0,271 | (0,156) |  | 15 | 0,266 | (0,117) | 0,255 | (0,121) |
| 17 | 0,727 | (0,246) | 0,268 | (0,107) |  | 17 | 0,635 | (0,214) | 0,27 | (0,111) |
| 20 | 0,183 | (0,07) | 0,227 | (0,12) |  | 20 | 0,18 | (0,065) | 0,218 | (0,103) |
| 23 | 0,242 | (0,113) | 0,366 | (0,265) |  | 23 | 0,226 | (0,131) | 0,397 | (0,292) |
| 25 | 0,596 | (0,201) | 0,302 | (0,07) |  | 25 | 0,633 | (0,233) | 0,317 | (0,079) |
| 26 | 0,649 | (0,308) | 0,229 | (0,089) |  | 26 | 0,633 | (0,239) | 0,237 | (0,101) |
| 45 | 0,355 | (0,126) | 0,296 | (0,092) |  | 45 | 0,322 | (0,113) | 0,296 | (0,089) |
| total | 0,52 | (0,137) | 0,287 | (0,08) |  | total | 0,532 | (0,159) | 0,295 | (0,08) |

Male sample table means of facial AU mean activation and mean intensity in the positive and negative story (N=25). Mean (SD).

| Negative Story | | | | |  | Positive story | | | | |
| --- | --- | --- | --- | --- | --- | --- | --- | --- | --- | --- |
| AU | Average intensity | | Average activation | |  | AU | Average intensity | | Average activation | |
| 1 | 0,297 | (0,167) | 0,248 | (0,098) |  | 1 | 0,289 | (0,155) | 0,284 | (0,092) |
| 2 | 0,11 | (0,043) | 0,305 | (0,102) |  | 2 | 0,128 | (0,069) | 0,313 | (0,096) |
| 4 | 0,643 | (0,554) | 0,327 | (0,337) |  | 4 | 0,698 | (0,585) | 0,317 | (0,325) |
| 5 | 0,077 | (0,029) | 0,732 | (0,331) |  | 5 | 0,078 | (0,036) | 0,71 | (0,309) |
| 6 | 0,723 | (0,44) | 0,191 | (0,228) |  | 6 | 0,803 | (0,455) | 0,217 | (0,229) |
| 7 | 1,698 | (0,864) | 0,266 | (0,283) |  | 7 | 1,785 | (0,798) | 0,301 | (0,278) |
| 9 | 0,107 | (0,05) | 0,137 | (0,126) |  | 9 | 0,106 | (0,043) | 0,118 | (0,096) |
| 10 | 1,065 | (0,439) | 0,287 | (0,289) |  | 10 | 1,108 | (0,469) | 0,326 | (0,339) |
| 12 | 0,284 | (0,327) | 0,132 | (0,23) |  | 12 | 0,31 | (0,318) | 0,114 | (0,145) |
| 14 | 0,577 | (0,391) | 0,29 | (0,261) |  | 14 | 0,588 | (0,389) | 0,284 | (0,261) |
| 15 | 0,349 | (0,171) | 0,3 | (0,127) |  | 15 | 0,339 | (0,2) | 0,329 | (0,119) |
| 17 | 0,649 | (0,154) | 0,219 | (0,076) |  | 17 | 0,619 | (0,199) | 0,261 | (0,081) |
| 20 | 0,223 | (0,114) | 0,287 | (0,114) |  | 20 | 0,212 | (0,108) | 0,266 | (0,123) |
| 23 | 0,202 | (0,079) | 0,365 | (0,311) |  | 23 | 0,195 | (0,059) | 0,37 | (0,284) |
| 25 | 0,562 | (0,195) | 0,282 | (0,072) |  | 25 | 0,569 | (0,159) | 0,262 | (0,083) |
| 26 | 0,626 | (0,164) | 0,241 | (0,089) |  | 26 | 0,638 | (0,184) | 0,228 | (0,072) |
| 45 | 0,27 | (0,084) | 0,286 | (0,101) |  | 45 | 0,268 | (0,096) | 0,293 | (0,105) |
| total | 0,498 | (0,128) | 0,274 | (0,076) |  | total | 0,514 | (0,126) | 0,279 | (0,071) |

**4) ST4. Partial correlations: male sample**

**mean intensity**

Positive story

| **AU** | **Affect** | **Initiation** | **Interest** | **Total** |
| --- | --- | --- | --- | --- |
| Mean intensity | Rho p | Rho p | Rho p | Rho p |
| **1** | -0,1045 | **-0,4725** | -0,3068 | -0,3740 |
|  | 0,6352 | 0,0228 | 0,1544 | 0,0787 |
| **2** | -0,0814 | -0,1338 | -0,0986 | -0,1283 |
|  | 0,7118 | 0,5426 | 0,6545 | 0,5597 |
| **4** | 0,0025 | 0,2073 | 0,2478 | 0,2328 |
|  | 0,9911 | 0,3426 | 0,2543 | 0,2851 |
| **5** | 0,0474 | 0,0364 | 0,0468 | 0,0831 |
|  | 0,8299 | 0,8692 | 0,8320 | 0,7063 |
| **6** | 0,0465 | 0,1761 | 0,3068 | 0,2325 |
|  | 0,8332 | 0,4216 | 0,1544 | 0,2858 |
| **7** | 0,0810 | -0,0207 | 0,1682 | 0,0889 |
|  | 0,7135 | 0,9255 | 0,4429 | 0,6865 |
| **9** | -0,3671 | -0,0834 | -0,0958 | -0,1683 |
|  | 0,0849 | 0,7052 | 0,6637 | 0,4427 |
| **10** | 0,0684 | -0,0575 | 0,0946 | 0,0495 |
|  | 0,7564 | 0,7943 | 0,6678 | 0,8225 |
| **12** | -0,3046 | -0,2858 | **-0,4178** | -0,4055 |
|  | 0,1576 | 0,1861 | 0,0473 | 0,0549 |
| **14** | -0,2311 | -0,3650 | -0,3683 | **-0,4159** |
|  | 0,2886 | 0,0868 | 0,0838 | 0,0484 |
| **15** | -0,3419 | -0,0171 | 0,2183 | 0,0307 |
|  | 0,1104 | 0,9382 | 0,3170 | 0,8893 |
| **17** | **-0,4369** | -0,1048 | 0,1278 | -0,0446 |
|  | 0,0371 | 0,6340 | 0,5611 | 0,8398 |
| **20** | -0,2750 | -0,1685 | -0,1195 | -0,1756 |
|  | 0,2040 | 0,4420 | 0,5869 | 0,4229 |
| **23** | 0,0531 | -0,3526 | -0,3539 | -0,3114 |
|  | 0,8097 | 0,0989 | 0,0976 | 0,1480 |
| **25** | -0,2533 | -0,2146 | -0,2195 | -0,2195 |
|  | 0,2436 | 0,3255 | 0,3142 | 0,3142 |
| **26** | -0,1728 | -0,2860 | -0,0723 | -0,2086 |
|  | 0,4305 | 0,1859 | 0,7431 | 0,3395 |
| **45** | -0,2889 | -0,3586 | -0,2465 | -0,3250 |
|  | 0,1812 | 0,0929 | 0,2569 | 0,1303 |

Negative story

| **AU** | **Affect** | **Initiation** | **Interest** | **Total** |
| --- | --- | --- | --- | --- |
| Mean intensity | Rho p | Rho p | Rho p | Rho p |
| **1** | -0,3303 | **-0,4320** | -0,2606 | **-0,4257** |
|  | 0,1237 | 0,0395 | 0,2298 | 0,0429 |
| **2** | -0,2644 | -0,0297 | 0,0137 | -0,1145 |
|  | 0,2228 | 0,8929 | 0,9504 | 0,6028 |
| **4** | 0,0851 | 0,2776 | 0,3653 | 0,3448 |
|  | 0,6994 | 0,1996 | 0,0866 | 0,1072 |
| **5** | -0,2140 | -0,0486 | 0,1423 | 0,0257 |
|  | 0,3268 | 0,8258 | 0,5170 | 0,9072 |
| **6** | 0,0053 | 0,1912 | 0,1293 | 0,1294 |
|  | 0,9809 | 0,3822 | 0,5567 | 0,5563 |
| **7** | -0,0289 | -0,1744 | -0,0118 | -0,0949 |
|  | 0,8958 | 0,4261 | 0,9574 | 0,6667 |
| **9** | -0,2723 | -0,3758 | -0,2566 | -0,3521 |
|  | 0,2088 | 0,0772 | 0,2373 | 0,0994 |
| **10** | -0,1177 | -0,0637 | 0,0291 | -0,0537 |
|  | 0,5926 | 0,7729 | 0,8953 | 0,8076 |
| **12** | -0,3141 | -0,1027 | -0,2298 | -0,2428 |
|  | 0,1443 | 0,6409 | 0,2915 | 0,2643 |
| **14** | -0,1534 | 0,0078 | -0,0842 | -0,0865 |
|  | 0,4848 | 0,9718 | 0,7025 | 0,6949 |
| **15** | **-0,6279** | -0,1772 | -0,0026 | -0,2295 |
|  | 0,0013 | 0,4186 | 0,9907 | 0,2922 |
| **17** | **-0,5105** | 0,0164 | 0,0364 | -0,0833 |
|  | 0,0128 | 0,9406 | 0,8691 | 0,7054 |
| **20** | **-0,4717** | -0,3999 | -0,2920 | -0,4102 |
|  | 0,0231 | 0,0587 | 0,1764 | 0,0519 |
| **23** | -0,1751 | -0,1985 | -0,0965 | -0,1480 |
|  | 0,4242 | 0,3638 | 0,6614 | 0,5004 |
| **25** | -0,2854 | -0,1519 | -0,3476 | -0,2796 |
|  | 0,1868 | 0,4889 | 0,1041 | 0,1964 |
| **26** | -0,1094 | -0,2818 | -0,1228 | -0,2550 |
|  | 0,6192 | 0,1926 | 0,5768 | 0,2403 |
| **45** | -0,2268 | -0,1875 | -0,2649 | -0,2645 |
|  | 0,2980 | 0,3916 | 0,2219 | 0,2226 |

**mean activation**

Positive story

| **AU** | **Affect** | **Initiation** | **Interest** | **Total** |
| --- | --- | --- | --- | --- |
| Mean activation | Rho p | Rho p | Rho p | Rho p |
| **1** | -0,2525 | -0,0131 | -0,0487 | -0,0864 |
|  | 0,2451 | 0,9528 | 0,8255 | 0,6949 |
| **2** | -0,1873 | 0,0102 | 0,0309 | -0,0230 |
|  | 0,3921 | 0,9632 | 0,8885 | 0,9170 |
| **4** | -0,0379 | 0,0452 | 0,0697 | 0,0859 |
|  | 0,8635 | 0,8377 | 0,7521 | 0,6969 |
| **5** | -0,0874 | 0,0207 | 0,0484 | -0,0106 |
|  | 0,6918 | 0,9254 | 0,8264 | 0,9617 |
| **6** | -0,0091 | -0,0830 | 0,0839 | 0,0140 |
|  | 0,9672 | 0,7066 | 0,7034 | 0,9495 |
| **7** | 0,0057 | -0,1170 | 0,1414 | 0,0228 |
|  | 0,9794 | 0,5948 | 0,5198 | 0,9177 |
| **9** | -0,3622 | -0,1520 | -0,3416 | -0,2992 |
|  | 0,0894 | 0,4887 | 0,1106 | 0,1655 |
| **10** | 0,1539 | -0,0868 | -0,1294 | -0,0362 |
|  | 0,4832 | 0,6936 | 0,5562 | 0,8699 |
| **12** | -0,2248 | -0,2228 | -0,3064 | -0,3068 |
|  | 0,3023 | 0,3069 | 0,1550 | 0,1545 |
| **14** | 0,1088 | **-0,5005** | **-0,6252** | **-0,5029** |
|  | 0,6211 | 0,0150 | 0,0014 | 0,0144 |
| **15** | -0,0667 | -0,0156 | 0,0690 | 0,0404 |
|  | 0,7622 | 0,9435 | 0,7544 | 0,8547 |
| **17** | 0,0083 | 0,0526 | 0,2872 | 0,1605 |
|  | 0,9702 | 0,8115 | 0,1839 | 0,4644 |
| **20** | -0,3309 | -0,1903 | -0,0814 | -0,2080 |
|  | 0,1230 | 0,3845 | 0,7121 | 0,3409 |
| **23** | 0,0632 | -0,0345 | -0,0356 | -0,0205 |
|  | 0,7747 | 0,8759 | 0,8720 | 0,9261 |
| **25** | -0,1337 | -0,1503 | -0,4111 | -0,2807 |
|  | 0,5430 | 0,4937 | 0,0513 | 0,1945 |
| **26** | 0,0988 | 0,0655 | -0,3115 | -0,0694 |
|  | 0,6536 | 0,7667 | 0,1479 | 0,7529 |
| **45** | -0,3762 | -0,3993 | **-0,4276** | **-0,4658** |
|  | 0,0768 | 0,0591 | 0,0418 | 0,0251 |

Negative story

| **AU** | **Affect** | **Initiation** | **Interest** | **Total** |
| --- | --- | --- | --- | --- |
| Mean activation | Rho p | Rho p | Rho p | Rho p |
| **1** | -0,1152 | -0,2184 | -0,1925 | -0,2480 |
|  | 0,6006 | 0,3169 | 0,3789 | 0,2539 |
| **2** | -0,0042 | -0,0901 | -0,0807 | -0,0950 |
|  | 0,9850 | 0,6826 | 0,7143 | 0,6663 |
| **4** | -0,1134 | 0,0225 | 0,2372 | 0,1170 |
|  | 0,6065 | 0,9189 | 0,2758 | 0,5949 |
| **5** | 0,1027 | 0,0306 | 0,1866 | 0,1293 |
|  | 0,6411 | 0,8897 | 0,3939 | 0,5566 |
| **6** | -0,0742 | -0,0831 | -0,0185 | -0,0768 |
|  | 0,7364 | 0,7061 | 0,9332 | 0,7275 |
| **7** | -0,1364 | -0,1280 | -0,0108 | -0,1081 |
|  | 0,5350 | 0,5606 | 0,9609 | 0,6236 |
| **9** | -0,2946 | -0,2245 | -0,1740 | -0,2609 |
|  | 0,1724 | 0,3031 | 0,4272 | 0,2292 |
| **10** | 0,0348 | -0,0899 | -0,1583 | -0,1012 |
|  | 0,8749 | 0,6833 | 0,4708 | 0,6461 |
| **12** | -0,1212 | 0,0945 | -0,0895 | -0,0410 |
|  | 0,5819 | 0,6681 | 0,6846 | 0,8527 |
| **14** | 0,0812 | -0,3005 | -0,3772 | -0,3222 |
|  | 0,7126 | 0,1636 | 0,0760 | 0,1338 |
| **15** | -0,2098 | 0,1434 | 0,2551 | 0,1083 |
|  | 0,3366 | 0,5141 | 0,2401 | 0,6229 |
| **17** | -0,1676 | 0,0222 | -0,0952 | -0,0800 |
|  | 0,4446 | 0,9201 | 0,6658 | 0,7168 |
| **20** | **-0,4736** | -0,0095 | 0,3021 | -0,0079 |
|  | 0,0225 | 0,9657 | 0,1612 | 0,9713 |
| **23** | -0,1439 | 0,0269 | -0,1445 | -0,0909 |
|  | 0,5125 | 0,9029 | 0,5106 | 0,6802 |
| **25** | -0,0693 | 0,3075 | -0,1127 | 0,0501 |
|  | 0,7534 | 0,1534 | 0,6085 | 0,8203 |
| **26** | 0,0043 | 0,1196 | -0,2141 | -0,0613 |
|  | 0,9843 | 0,5868 | 0,3265 | 0,7813 |
| **45** | -0,1308 | -0,0188 | 0,1487 | 0,0631 |
|  | 0,5520 | 0,9323 | 0,4983 | 0,7749 |

**5) Partial correlations : Female sample**

**mean intensity**

Positive story

| **AU** | **Affect** | **Initiation** | **Interest** | **Total** |
| --- | --- | --- | --- | --- |
| Mean intensity | Rho p | Rho p | Rho p | Rho p |
| **1** | -0,2128 | 0,2260 | 0,0293 | 0,1864 |
|  | 0,2128 | 0,1850 | 0,8653 | 0,2765 |
| **2** | -0,2970 | 0,0720 | -0,2247 | -0,0690 |
|  | 0,0785 | 0,6764 | 0,1876 | 0,6893 |
| **4** | -0,0407 | 0,0285 | -0,1524 | -0,0443 |
|  | 0,8138 | 0,8691 | 0,3750 | 0,7974 |
| **5** | -0,0997 | 0,0732 | -0,1743 | -0,0103 |
|  | 0,5631 | 0,6715 | 0,3093 | 0,9523 |
| **6** | -0,0178 | 0,0158 | 0,0710 | 0,0071 |
|  | 0,9180 | 0,9273 | 0,6808 | 0,9670 |
| **7** | -0,1179 | 0,1117 | 0,1883 | 0,1428 |
|  | 0,4935 | 0,5165 | 0,2715 | 0,4060 |
| **9** | -0,2320 | 0,0136 | 0,0075 | 0,0563 |
|  | 0,1734 | 0,9373 | 0,9655 | 0,7442 |
| **10** | -0,3095 | -0,2607 | -0,1761 | -0,2044 |
|  | 0,0663 | 0,1246 | 0,3041 | 0,2319 |
| **12** | -0,2280 | -0,1978 | -0,1904 | -0,2473 |
|  | 0,1810 | 0,2475 | 0,2660 | 0,1459 |
| **14** | -0,1225 | 0,0472 | 0,0318 | -0,0502 |
|  | 0,4765 | 0,7844 | 0,8541 | 0,7713 |
| **15** | -0,0996 | 0,0322 | -0,2237 | -0,0669 |
|  | 0,5634 | 0,8523 | 0,1896 | 0,6984 |
| **17** | -0,2454 | 0,0709 | 0,0407 | 0,0872 |
|  | 0,1492 | 0,6812 | 0,8139 | 0,6131 |
| **20** | -0,0759 | 0,2463 | 0,0810 | 0,2238 |
|  | 0,6598 | 0,1476 | 0,6385 | 0,1894 |
| **23** | -0,0855 | 0,2915 | 0,1076 | 0,2840 |
|  | 0,6200 | 0,0846 | 0,5324 | 0,0933 |
| **25** | -0,2447 | -0,1009 | -0,0764 | -0,1331 |
|  | 0,1504 | 0,5580 | 0,6579 | 0,4391 |
| **26** | -0,1397 | -0,0324 | -0,2048 | -0,1026 |
|  | 0,4163 | 0,8511 | 0,2309 | 0,5517 |
| **45** | -0,2690 | 0,0510 | -0,1163 | 0,0251 |
|  | 0,1127 | 0,7676 | 0,4994 | 0,8843 |

Negative story

| **AU** | **Affect** | **Initiation** | **Interest** | **Total** |
| --- | --- | --- | --- | --- |
| mean intensity | Rho p | Rho p | Rho p | Rho p |
| **1** | **-0,3607** | 0,1523 | 0,0704 | 0,2019 |
|  | 0,0307 | 0,3752 | 0,6831 | 0,2378 |
| **2** | -0,1454 | -0,0382 | -0,2428 | -0,1110 |
|  | 0,3975 | 0,8250 | 0,1536 | 0,5194 |
| **4** | -0,1049 | 0,0573 | -0,1225 | -0,0395 |
|  | 0,5427 | 0,7400 | 0,4767 | 0,8191 |
| **5** | -0,0886 | 0,1533 | 0,0686 | 0,2161 |
|  | 0,6072 | 0,3722 | 0,6909 | 0,2057 |
| **6** | 0,1707 | -0,0719 | 0,0553 | -0,0294 |
|  | 0,3197 | 0,6770 | 0,7489 | 0,8646 |
| **7** | 0,0504 | -0,0896 | 0,1669 | 0,0737 |
|  | 0,7702 | 0,6031 | 0,3305 | 0,6693 |
| **9** | **-0,4696** | -0,0170 | -0,2332 | -0,1376 |
|  | 0,0039 | 0,9215 | 0,1711 | 0,4236 |
| **10** | -0,2239 | -0,3287 | -0,1444 | -0,2003 |
|  | 0,1893 | 0,0503 | 0,4007 | 0,2415 |
| **12** | -0,1911 | -0,3168 | -0,2013 | -0,2934 |
|  | 0,2641 | 0,0598 | 0,2391 | 0,0824 |
| **14** | -0,1211 | 0,0101 | 0,1086 | 0,0250 |
|  | 0,4817 | 0,9536 | 0,5282 | 0,8851 |
| **15** | -0,2124 | -0,1236 | -0,2316 | -0,1564 |
|  | 0,2136 | 0,4728 | 0,1741 | 0,3623 |
| **17** | -0,1912 | -0,1083 | -0,1532 | -0,1193 |
|  | 0,2640 | 0,5297 | 0,3724 | 0,4883 |
| **20** | -0,3107 | -0,0209 | -0,1852 | -0,1072 |
|  | 0,0652 | 0,9039 | 0,2795 | 0,5338 |
| **23** | -0,1821 | 0,0204 | -0,2150 | -0,0348 |
|  | 0,2877 | 0,9061 | 0,2079 | 0,8401 |
| **25** | -0,2137 | -0,0909 | -0,0711 | -0,0751 |
|  | 0,2109 | 0,5982 | 0,6803 | 0,6635 |
| **26** | -0,1957 | 0,0848 | -0,1675 | 0,0214 |
|  | 0,2526 | 0,6231 | 0,3288 | 0,9016 |
| **45** | -0,2038 | 0,0570 | -0,1084 | -0,0108 |
|  | 0,2331 | 0,7410 | 0,5291 | 0,9504 |

**Mean activation**

Positive story

| **AU** | **Affect** | **Initiation** | **Interest** | **Total** |
| --- | --- | --- | --- | --- |
| Mean activation | Rho p | R p | Rho p | Rho p |
| **1** | -0,0179 | 0,2411 | 0,1859 | 0,3032 |
|  | 0,9175 | 0,1566 | 0,2778 | 0,0723 |
| **2** | -0,0462 | 0,1066 | -0,0120 | 0,1536 |
|  | 0,7892 | 0,5359 | 0,9448 | 0,3712 |
| **4** | -0,2901 | -0,2560 | -0,1084 | -0,2064 |
|  | 0,0862 | 0,1318 | 0,5293 | 0,2272 |
| **5** | 0,2676 | 0,0315 | 0,1110 | 0,1206 |
|  | 0,1145 | 0,8555 | 0,5193 | 0,4836 |
| **6** | -0,1049 | 0,0242 | 0,1685 | 0,0937 |
|  | 0,5427 | 0,8887 | 0,3258 | 0,5869 |
| **7** | -0,2533 | 0,0212 | 0,0701 | -0,0066 |
|  | 0,1361 | 0,9024 | 0,6847 | 0,9694 |
| **9** | 0,0971 | 0,0860 | 0,0186 | 0,1103 |
|  | 0,5734 | 0,6178 | 0,9142 | 0,5218 |
| **10** | **-0,4615** | -0,3178 | -0,0770 | -0,2051 |
|  | 0,0046 | 0,0589 | 0,6555 | 0,2302 |
| **12** | -0,0044 | -0,1266 | -0,1267 | -0,1981 |
|  | 0,9797 | 0,4620 | 0,4615 | 0,2467 |
| **14** | -0,0332 | -0,0520 | 0,0132 | -0,0534 |
|  | 0,8474 | 0,7633 | 0,9392 | 0,7573 |
| **15** | -0,1775 | 0,0420 | -0,1553 | -0,0353 |
|  | 0,3005 | 0,8078 | 0,3658 | 0,8382 |
| **17** | -0,0085 | 0,0392 | -0,0010 | 0,0077 |
|  | 0,9610 | 0,8206 | 0,9955 | 0,9644 |
| **20** | -0,1263 | 0,1898 | -0,1603 | 0,0697 |
|  | 0,4629 | 0,2675 | 0,3503 | 0,6864 |
| **23** | 0,1395 | 0,0706 | 0,1986 | 0,1857 |
|  | 0,4171 | 0,6822 | 0,2456 | 0,2782 |
| **25** | **-0,3332** | -0,0262 | 0,0444 | -0,0269 |
|  | 0,0471 | 0,8794 | 0,7971 | 0,8763 |
| **26** | -0,1708 | -0,1430 | -0,1529 | -0,1538 |
|  | 0,3192 | 0,4054 | 0,3732 | 0,3706 |
| **45** | -0,2061 | -0,0252 | -0,1386 | -0,0791 |
|  | 0,2278 | 0,8839 | 0,4202 | 0,6467 |

Negative story

| **AU** | **Affect** | **Initiation** | **Interest** | **Total** |
| --- | --- | --- | --- | --- |
| Mean activation | Rho p | Rho p | Rho p | Rho p |
| **1** | **-0,3403** | 0,2046 | -0,0934 | 0,0201 |
|  | 0,0422 | 0,2313 | 0,5880 | 0,9072 |
| **2** | -0,4221 | -0,0139 | -0,2918 | -0,1305 |
|  | 0,0103 | 0,9361 | 0,0842 | 0,4482 |
| **4** | -0,3108 | -0,2126 | -0,0826 | -0,1833 |
|  | 0,0650 | 0,2131 | 0,6321 | 0,2846 |
| **5** | 0,2991 | -0,0093 | -0,0044 | -0,0122 |
|  | 0,0763 | 0,9573 | 0,9795 | 0,9436 |
| **6** | 0,0266 | -0,1477 | -0,0139 | -0,0911 |
|  | 0,8776 | 0,3901 | 0,9357 | 0,5974 |
| **7** | 0,0241 | -0,1333 | 0,0127 | -0,0621 |
|  | 0,8890 | 0,4382 | 0,9412 | 0,7190 |
| **9** | **-0,3816** | 0,0717 | -0,1410 | 0,0154 |
|  | 0,0216 | 0,6779 | 0,4119 | 0,9291 |
| **10** | **-0,3501** | **-0,4264** | -0,2133 | **-0,3369** |
|  | 0,0363 | 0,0095 | 0,2116 | 0,0445 |
| **12** | -0,1159 | -0,2433 | -0,2829 | **-0,3495** |
|  | 0,5010 | 0,1528 | 0,0946 | 0,0367 |
| **14** | -0,1251 | -0,0795 | -0,0675 | -0,0804 |
|  | 0,4671 | 0,6451 | 0,6958 | 0,6409 |
| **15** | -0,0955 | 0,0312 | -0,0421 | 0,0617 |
|  | 0,5795 | 0,8568 | 0,8074 | 0,7207 |
| **17** | -0,2435 | -0,2144 | -0,0696 | -0,2138 |
|  | 0,1524 | 0,2092 | 0,6867 | 0,2106 |
| **20** | -0,1201 | -0,1173 | -0,1759 | -0,1534 |
|  | 0,4853 | 0,4958 | 0,3049 | 0,3716 |
| **23** | 0,0145 | -0,0501 | 0,1002 | 0,0600 |
|  | 0,9329 | 0,7718 | 0,5610 | 0,7279 |
| **25** | -0,0225 | 0,0523 | -0,0052 | 0,0232 |
|  | 0,8965 | 0,7618 | 0,9761 | 0,8930 |
| **26** | -0,1489 | 0,1055 | -0,2858 | -0,1225 |
|  | 0,3861 | 0,5405 | 0,0911 | 0,4766 |
| **45** | **-0,3490** | -0,0071 | 0,0353 | -0,0003 |
|  | 0,0370 | 0,9671 | 0,8378 | 0,9986 |
